# RETRACTION: Researching How Music Affects the Autonomic Nervous System and Influences Wound Healing Processes in Trauma Patients

**DOI:** 10.1111/iwj.70499

**Published:** 2025-04-02

## Abstract

**RETRACTION:** LiX. , and minS. , “Researching How Music Affects the Autonomic Nervous System and Influences Wound Healing Processes in Trauma Patients,” International Wound Journal 21, no. 3 (2024): e14790, 10.1111/iwj.14790.38414351 PMC10899861

The above article, published online on 27 February 2024, in Wiley Online Library (http://onlinelibrary.wiley.com/), has been retracted by agreement between the journal Editor in Chief, Professor Keith Harding; and John Wiley & Sons Ltd. Following an investigation by the publisher, all parties have concluded that this article was accepted solely on the basis of a compromised peer review process. The editors have therefore decided to retract the article. The authors did not respond to our notice regarding the retraction.

**RETRACTION:** X. Li , and S. min , “Researching How Music Affects the Autonomic Nervous System and Influences Wound Healing Processes in Trauma Patients,” International Wound Journal 21, no. 3 (2024): e14790, 10.1111/iwj.14790.38414351 PMC10899861

The above article, published online on 27 February 2024, in Wiley Online Library (http://onlinelibrary.wiley.com/), has been retracted by agreement between the journal Editor in Chief, Professor Keith Harding; and John Wiley & Sons Ltd. Following an investigation by the publisher, all parties have concluded that this article was accepted solely on the basis of a compromised peer review process. The editors have therefore decided to retract the article. The authors did not respond to our notice regarding the retraction.

